# Pleiotrophin promotes chemoresistance to doxorubicin in osteosarcoma by upregulating P-glycoprotein

**DOI:** 10.18632/oncotarget.19148

**Published:** 2017-07-10

**Authors:** Dapeng Wu, Liguo Liu, Xuebing Yan, Chunyan Wang, Yaling Wang, Kun Han, Shuchen Lin, Zhihua Gan, Daliu Min

**Affiliations:** ^1^ Department of Oncology, Shanghai Jiao Tong University Affiliated Sixth People's Hospital, Shanghai, China; ^2^ Department of General Surgery, Shanghai Jiao Tong University Affiliated Sixth People's Hospital, Shanghai, China; ^3^ Department of General Surgery, Shanghai Tenth People's Hospital, Tongji University School of Medicine, Shanghai, China

**Keywords:** PTN, osteosarcoma, chemoresistance, doxorubicin, β-catenin

## Abstract

Chemoresistance is a major hindrance to successful treatment of osteosarcoma (OS). Pleiotrophin (PTN), a neurotrophic growth factor, has been linked to the malignant characteristics of various cancer types. We retrospectively examined the correlation between PTN expression and chemoresistance in OS in a cohort of 133 OS patients. Immunohistochemistry revealed that PTN expression correlated with the necrosis rate and local OS recurrence. In a prognostic analysis, high PTN expression was associated with poor overall and disease-free survival, and was an independent adverse prognostic factor for disease-free survival. In doxorubicin-treated OS cells, PTN knockdown enhanced cellular chemosensitivity, increased the apoptosis rate and inhibited clone formation, while PTN overexpression had the opposite effects. In a xenograft model, PTN knockdown and overexpression respectively enhanced and reduced cellular sensitivity to doxorubicin. PTN upregulated anaplastic lymphoma kinase (ALK), p-Glycogen Synthase Kinase (GSK)3β, β-catenin and multidrug resistance protein 1/P-glycoprotein (MDR1/P-gp). In rescue assays with the β-catenin inhibitor XAV939 and the MDR1/P-gp inhibitor verapamil, PTN promoted chemoresistance to doxorubicin in OS cells by activating ALK/GSK3β/β-catenin signaling, thereby upregulating MDR1/P-gp. Therefore, PTN could be used as a biomarker predicting chemotherapeutic responses, and downregulating PTN could be a promising therapeutic strategy to prevent chemoresistance in OS patients.

## INTRODUCTION

Osteosarcoma (OS) is the most common solid bone malignancy and the second leading cause of cancer-related death in children and adolescents. This highly aggressive cancer has often metastasized by the time it is diagnosed [1]. Due to the implementation of multi-agent neoadjuvant and adjuvant chemotherapy in the 1970s, the 5-year survival rate of patients with OS has dramatically increased from approximately 10% to 70% [2]. However, this encouraging breakthrough has not been completely translated into improved clinical outcomes in OS patients, largely due to chemoresistance during treatment [3].

Recent studies have highlighted the molecular changes that promote OS chemoresistance, such as abnormal expression of multidrug resistance-associated proteins, DNA Topo II, Apurinic endonuclease 1, microRNAs and long noncoding RNAs, but the existing data are far from sufficient [4, 5]. Therefore, there is an urgent need to elucidate the molecular pathways underlying OS chemoresistance and to identify biomarkers that not only predict individual chemotherapy responses, but also can be effectively targeted to overcome chemoresistance.

Pleiotrophin (PTN) is a heparin-binding neurotrophic growth factor with diverse biological functions, including angiogenesis, cellular differentiation and proliferation [6]. PTN has been implicated in the tumorigenesis of numerous human malignancies, including colorectal cancer, glioblastoma, melanoma, pancreatic cancer, breast cancer and lung cancer [7–12]. For example, PTN promotes angiogenesis by upregulating vascular endothelial growth factor in colorectal cancer [7]. PTN also contributes to platelet-derived-growth-factor-B-induced gliomagenesis by enhancing the proliferation of neural progenitor cells [8]. Furthermore, retrospective studies based on clinical specimens have demonstrated the potential of PTN as a diagnostic and prognostic biomarker for cancer patients [13–15].

Regarding OS, a microarray analysis by Mintz et al. demonstrated that *PTN* expression was higher in OS patients responding poorly to chemotherapy than in those responding well to chemotherapy [16]. Using the same method, Walters et al. subsequently demonstrated that *PTN* expression was dramatically greater in chemoresistant cell lines than in their parental cell lines [17]. However, following the above studies, no further efforts have been made to investigate the specific biological function and relevant molecular mechanism of PTN in OS chemoresistance. Furthermore, it remains unknown whether PTN is a predictive biomarker of patient survival or chemotherapeutic responses in OS.

Therefore, in this study, we investigated the clinical significance of PTN in a retrospective cohort study of 133 OS patients who received radical surgery combined with standard chemotherapy. Then, we performed functional assays *in vitro* and *in vivo* to determine whether PTN influences the doxorubicin (DOX) sensitivity of OS cells. Finally, we investigated the molecular pathway whereby PTN affects the cellular sensitivity to DOX.

## RESULTS

### Expression and prognostic significance of PTN in OS patients

**Figure 1:**
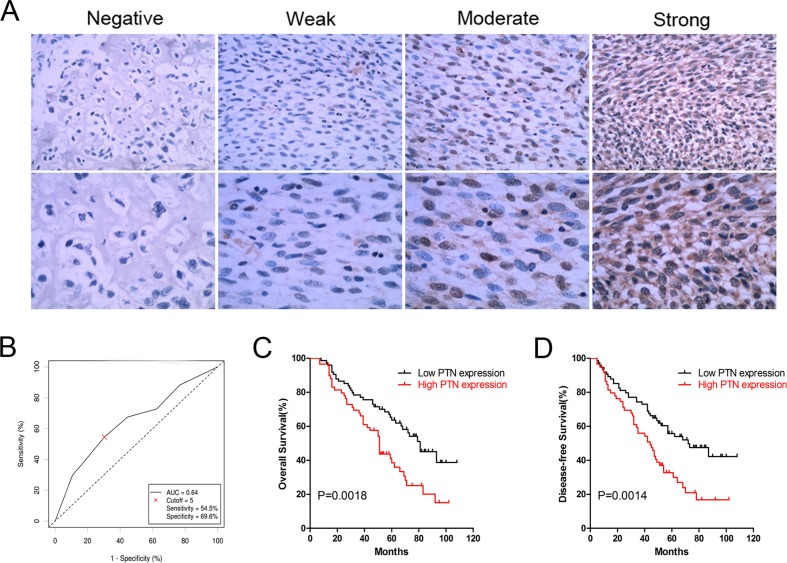
Expression and prognostic significance of PTN in OS patients **(A)** Representative immunohistochemical staining of PTN in OS tissues at 200× (upper) and 400× (lower) magnification. **(B)** The ROC curve for the IRS was employed to determine the cutoff value of PTN expression. **(C)** Overall survival curves of OS patients stratified by PTN expression. **(D)** DFS curves of OS patients stratified by PTN expression. PTN: pleiotrophin; OS: osteosarcoma; ROC: receiver operator characteristics.

PTN expression was evaluated in OS samples from patients; representative results are shown in Figure 1A. PTN was mainly detected in the cytoplasm of OS cells, and receiver operating characteristic (ROC) curve analysis indicated that the cutoff value of the immunoreactive score (IRS) was 5 (Figure 1B). Thus, we used this cutoff value to divide the patients into a high expression group (IRS≥6, n=59) and a low expression group (IRS<6, n=74).

The correlations between PTN expression and clinical features in OS patients are shown in Table 1. PTN expression correlated significantly with the necrosis rate (p=0.003) and local tumor recurrence (p=0.001), but not with other clinical features including gender (p=0.924), age (p=0.274), tumor location (p=0.472), lung metastasis (p=0.109) and TNM stage (p=0.135). Kaplan-Meier survival analysis indicated that high PTN expression correlated strongly with worse overall survival (p=0.0018, Figure 1C) and disease-free survival (DFS) in OS patients (p=0.0014, Figure 1D). As shown in Tables 2 and 3, univariate analysis indicated that PTN expression (high *vs*. low), the TNM stage (III+IV *vs*. I+II) and the necrosis rate (<90% *vs*. ≥90%) were significant factors influencing the overall survival and DFS of patients (overall survival: p=0.002, p<0.001 and p<0.001; DFS: p=0.002, p<0.001 and p<0.001, respectively). However, in multivariate analysis, the TNM stage and necrosis rate were independent prognostic factors for overall survival (p<0.001 and p=0.01, respectively), while PTN expression, the TNM stage and the necrosis rate were independent prognostic factors for DFS (p=0.049, p<0.001 and p=0.006).

**Table 1:** Relationship between PTN expression and clinicopathologic factors in OS patients

Variables	Total	PTN expression		Chi-square	P value
	(n=133)	Low	High		
Gender					
Male	84	47	37	0.009	0.924
Female	49	27	22		
Age (years)					
≤20	88	46	42	1.194	0.274
>20	45	28	17		
Necrosis Rate					
Low (<90%)	73	32	41	9.134	**0.003**
High (≥90%)	60	42	18		
Tumor Location					
Femur	70	40	30	3.536	0.472
Tibia	47	24	23		
Humerus	10	6	4		
Fibula	3	3	0		
Other sites	3	1	2		
Local Recurrence					
Yes	64	26	38	11.267	**0.001**
No	69	48	21		
Lung Metastasis					
Yes	38	17	21	2.562	0.109
No	95	57	38		
TNM Stage					
I/II	66	41	25	2.23	0.135
III/IV	67	33	34		

PTN: pleiotrophin; OS: osteosarcoma.

**Table 2:** Univariate and multivariate analyses of prognostic factors for overall survival

Variables	Univariate analysis			Multivariate analysis		
	HR	95%CI	P value	HR	95%CI	P value
Gender	0.846	0.532-1.344	0.479	0.623	0.380-1.022	0.061
Age	0.857	0.531-1.383	0.528	0.974	0.588-1.613	0.917
Tumor location	1.078	0.855-1.360	0.524	1.019	0.799-1.299	0.88
PTN expression	2.014	1.280-3.170	**0.002**	1.584	0.982-2.553	0.059
Necrosis rate	3.246	1.955-5.390	**<0.001**	2.098	1.193-3.691	**0.01**
TNM stage	3.738	2.272-6.150	**<0.001**	3.016	1.768-5.145	**<0.001**

PTN: pleiotrophin; HR: hazard ratio; CI: confidence interval.

**Table 3:** Univariate and multivariate analyses of prognostic factors for disease-free survival

Variables	Univariate analysis			Multivariate analysis		
	HR	95%CI	P value	HR	95%CI	P value
Gender	0.893	0.568-1.403	0.623	1.684	0.425-1.102	0.119
Age	0.876	0.549-1.397	0.577	1.026	0.628-1.675	0.920
Tumor location	1.066	0.846-1.343	0.590	0.997	0.786-1.266	0.983
PTN expression	2.018	1.297-3.142	**0.002**	1.600	1.002-2.555	**0.049**
Necrosis rate	3.121	1.917-5.082	**<0.001**	2.155	1.253-3.707	**0.006**
TNM stage	3.361	2.100-5.379	**<0.001**	2.699	1.636-4.452	**<0.001**

PTN: pleiotrophin; HR: hazard ratio; CI: confidence interval.

### Expression of PTN and chemoresistance to DOX in OS cell lines

**Figure 2:**
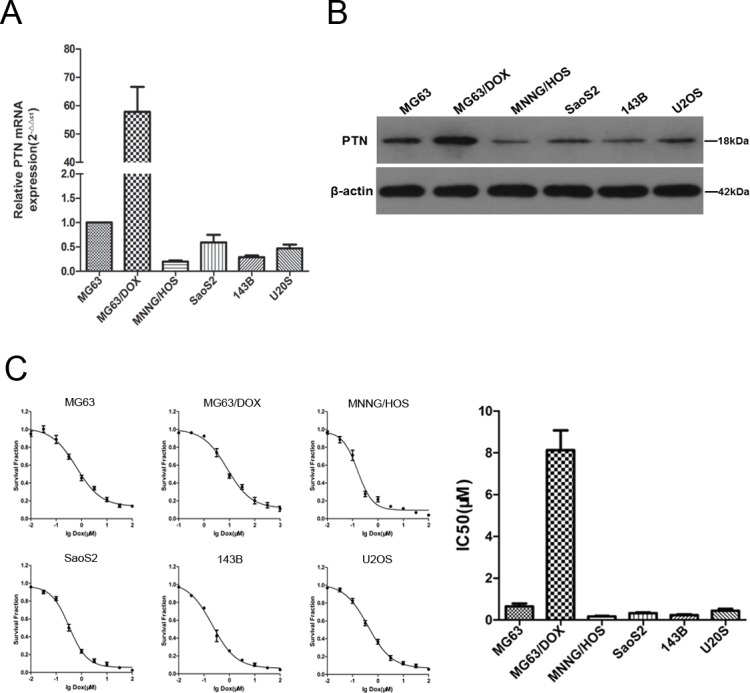
Expression of PTN and chemoresistance to DOX in OS cell lines **(A)** The mRNA levels of *PTN* in OS cell lines were analyzed by quantitative RT-PCR (*β-actin* was used as the reference gene and *PTN* expression in MG63 cells was used as the baseline). **(B)** The protein levels of PTN in OS cell lines were analyzed by Western blotting (β-actin was used as the reference protein). **(C)** The IC50 of DOX was determined by the CCK-8 assay in OS cell lines. PTN: pleiotrophin; OS: osteosarcoma; DOX: doxorubicin; IC50: half maximal inhibitory concentration; CCK-8: Cell Counting Kit-8.

Next, RT-PCR was performed to detect the mRNA expression of *PTN* in several commonly used OS cell lines, including MNNG/HOS, SaoS2, 143B, U2OS, MG63 and its DOX-resistant subline (MG63/DOX). As shown in Figure 2A, the mRNA expression of *PTN* was significantly higher in the DOX-resistant MG63/DOX subline than in the other cell lines, including MNNG/HOS, SaoS2, 143B and U2OS cells and the parental MG63 cells. Western blotting was performed, and confirmed the RT-PCR results (Figure 2B). Furthermore, a similar tendency was observed for the DOX sensitivity of OS cells, as the half-maximal inhibitory concentration (IC50) was higher in the DOX-resistant MG63/DOX subline than in the other cell lines (shown in Figure 2C). Taken together, these findings suggested a potential association between PTN expression and DOX resistance in OS cells.

### PTN promotes cellular resistance to DOX *in vitro*

**Figure 3:**
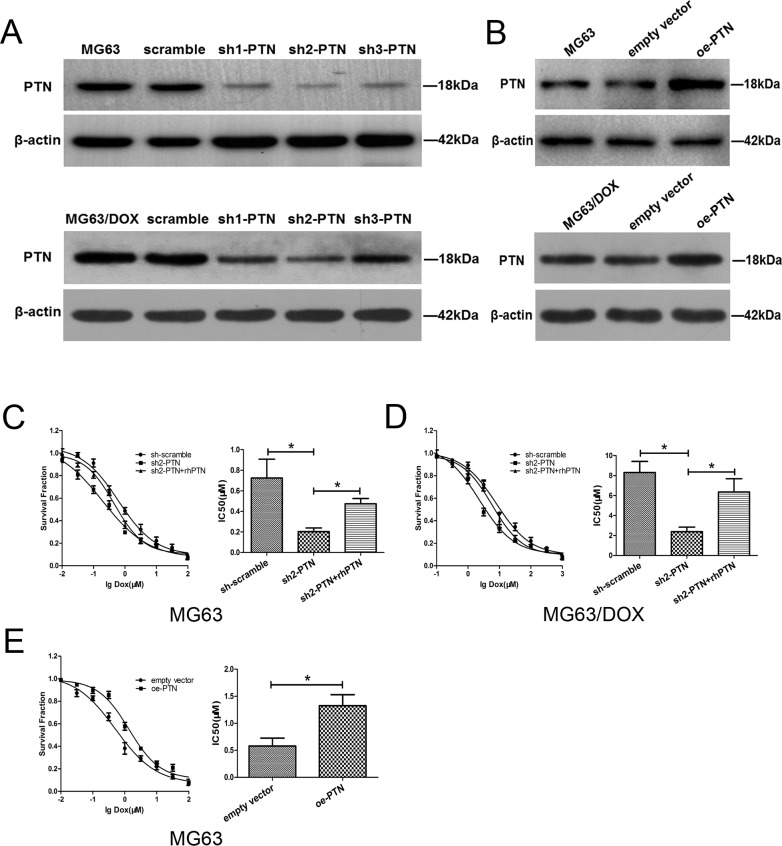
PTN promotes OS resistance to DOX *in vitro* **(A)** The knockdown efficiencies of three shRNAs targeting *PTN* were examined in MG63 (upper) and MG63/DOX (lower) cells by Western blotting. **(B)** The overexpression efficiency of the plasmid was examined in MG63 (upper) and MG63/DOX (lower) cells by Western blotting. **(C, D)** PTN knockdown prevented DOX resistance in MG63 (C) and MG63/DOX (D) cells, while supplementation with rhPTN for 24 h (100 ng/mL) could reverse this effect. **(E)** PTN overexpression promoted DOX resistance in MG63 cells. *p < 0.05. PTN: pleiotrophin; OS: osteosarcoma; DOX: doxorubicin; rhPTN: recombinant human pleiotrophin; sh: short hairpin; oe: overexpression.

To further investigate the involvement of PTN in OS chemoresistance *in vitro* and *in vivo*, we used three short hairpin (sh) RNAs to downregulate PTN expression in MG63, MG63/DOX and U2OS cells and evaluated their knockdown efficiency by Western blotting (Figure 3A and Supplementary Figure 1A). As sh2-PTN appeared to have the best knockdown efficiency, we selected it for subsequent assays. Moreover, a plasmid carrying *PTN* cDNA was employed to upregulate PTN expression in these OS cells, and its overexpression efficiency was validated by Western blotting (Figure 3B and Supplementary Figure 1B).

A chemosensitivity assay was then performed to investigate whether PTN expression influences DOX resistance in OS cells. As shown in Figures 3C and 3D, MG63 and MG63/DOX cells both became more sensitive to DOX treatment after PTN was downregulated (all p<0.05). However, this effect could be reversed in both types of cells by incubation with 100 ng/mL recombinant human PTN (rhPTN) for 24 h (all p<0.05). Similar results were observed in U2OS cells (all p<0.05, Supplementary Figure 1C). Moreover, Figure 3E and Supplementary Figure 1D demonstrate that the upregulation of PTN significantly enhanced DOX resistance in both MG63 and U2OS cells (all p<0.05). We were unable to further upregulate PTN expression in MG63/DOX cells for the chemosensitivity assay, largely because PTN expression is already extremely high in these cells, as shown in Figures 2A and 2B. Nevertheless, these findings strongly supported our hypothesis that PTN promotes DOX resistance in OS cells.

### PTN promotes apoptosis resistance and colony formation in OS cells treated with DOX

**Figure 4:**
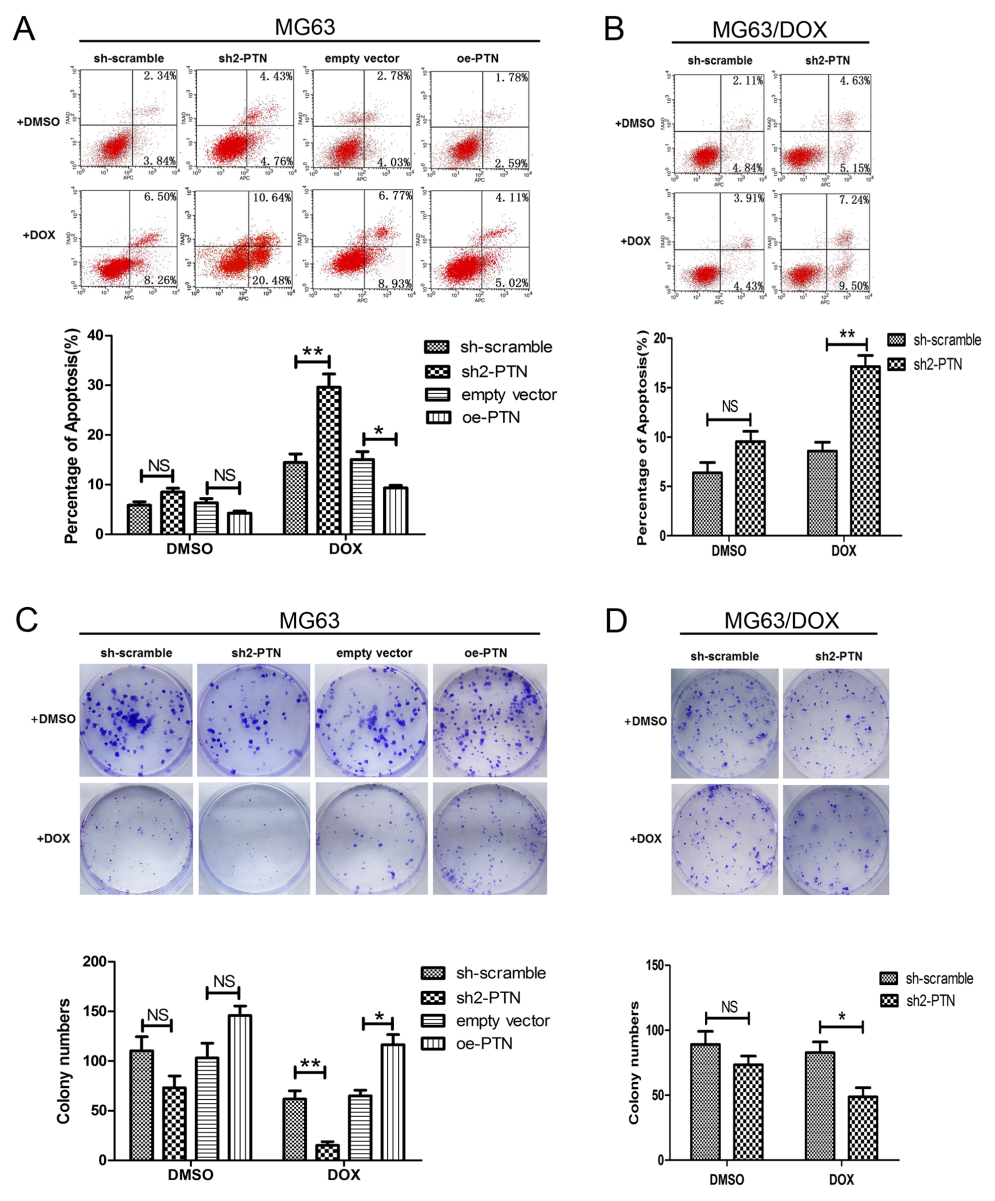
PTN promotes apoptosis resistance and colony formation in OS cells treated with DOX **(A, B)** PTN expression had no effect on the apoptosis of MG63 (A) and MG63/DOX (B) cells treated with DMSO. PTN knockdown promoted apoptosis in MG63 (A) and MG63/DOX (B) cells treated with DOX, while PTN overexpression inhibited apoptosis in MG63 cells treated with DOX. **(C, D)** PTN expression had no effect on the clone formation of MG63 (C) and MG63/DOX (D) cells treated with DMSO. PTN knockdown inhibited the clone formation of MG63 (C) and MG63/DOX (D) cells treated with DOX, while PTN overexpression promoted the clone formation of MG63 cells treated with DOX. *, p<0.05; **, p<0.01. PTN: pleiotrophin; OS: osteosarcoma; DOX: doxorubicin; sh: short hairpin; oe: overexpression. NS: not significant.

The impact of PTN on DOX-induced apoptosis was then analyzed by flow cytometry. As shown in Figures 4A and 4B, after cells were incubated with 0.1 μM DOX for 48 h, PTN knockdown increased the apoptosis rates of MG63 and MG63/DOX cells, while PTN overexpression reduced the apoptosis rate of MG63 cells (all p<0.05). No significant effect on DMSO-induced apoptosis was observed for either OS cell type after PTN expression was altered (all p>0.05). Similar effects of PTN on DOX-induced apoptosis were observed in U2OS cells (all p<0.05, Supplementary Figure 2A).

The effect of PTN on the clonogenic abilities of OS cells treated with DOX was analyzed by colony formation analysis (Figures 4C and 4D). PTN knockdown significantly inhibited the clonogenicity of MG63 and MG63/DOX cells treated with DOX, while PTN overexpression had the opposite effect in MG63 cells (all p<0.05). Although an inconspicuous difference in the colony formation of DMSO-treated OS cells was observed after PTN expression was altered, the results were not statistically significant (all p>0.05). However, PTN did enhance the clonogenicity of U2OS cells after DOX treatment, as shown in Supplementary Figure 2B (all p<0.05).

### PTN promotes OS resistance to DOX *in vivo*

**Figure 5:**
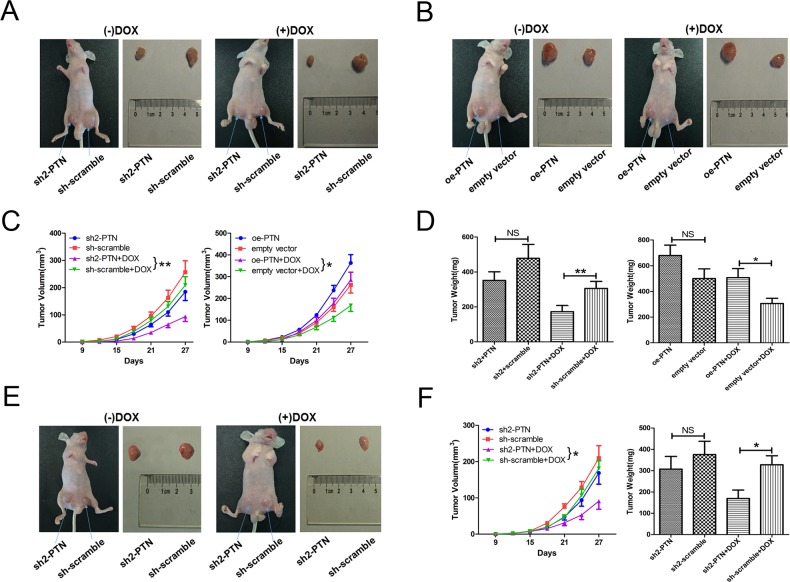
PTN promotes OS resistance to DOX *in vivo* **(A)** Representative images of nude mice treated with normal saline or DOX (left) and harvested xenografts formed by stable PTN-knockdown or scrambled-control MG63 cells (right). **(B)** Representative images of nude mice treated with normal saline or DOX (left) and harvested xenografts formed by stable PTN-overexpressing or empty-vector-control MG63 cells (right). **(C)** Time-dependent changes in the tumor volumes of MG63 cells with PTN knockdown (left) or overexpression (right). **(D)** The final weights of harvested tumors of MG63 cells with PTN knockdown (left) and overexpression (right). **(E)** Representative images of nude mice treated with normal saline or DOX (left) and harvested xenografts formed by stable PTN-knockdown or scrambled-control MG63/DOX cells (right). **(F)** Time-dependent volume changes (left) and final weights (right) of harvested tumorsof MG63/DOX cells. *, p<0.05; **, p<0.01. PTN: pleiotrophin; OS: osteosarcoma; DOX: doxorubicin; sh: short hairpin; oe: overexpression; NS: not significant.

To determine whether PTN promotes OS resistance to DOX *in vivo*, we subcutaneously injected BALB/c-nu mice with PTN-knockdown/overexpressing MG63 cells. DOX treatment was performed nine days after the cell injection. Representative images of the xenograft model and matched harvested tumors are shown in Figures 5A (PTN knockdown) and 5B (PTN overexpression). After DOX treatment, PTN knockdown significantly inhibited xenograft tumor growth (left in Figure 5C, p=0.009), while PTN overexpression enhanced tumor growth (right in Figure 5C, p=0.025). This result was further confirmed when the final tumor weights of each group were compared (Figure 5D, all p<0.05). Although up- or downregulating PTN expression slightly altered the growth and weights of tumors in the groups treated with normal saline, the results were statistically insignificant (all p>0.05). Furthermore, as shown in Figures 5E and 5F, knocking down PTN in MG63/DOX cells also increased DOX sensitivity *in vivo* (tumor growth: p=0.015; tumor weight: p=0.021). Taken together, these results firmly support the notion that PTN also promotes DOX resistance in OS *in vivo*.

### PTN promotes OS resistance to DOX by upregulating MDR1/P-gp through the ALK/GSK3β/β-catenin signaling pathway

**Figure 6:**
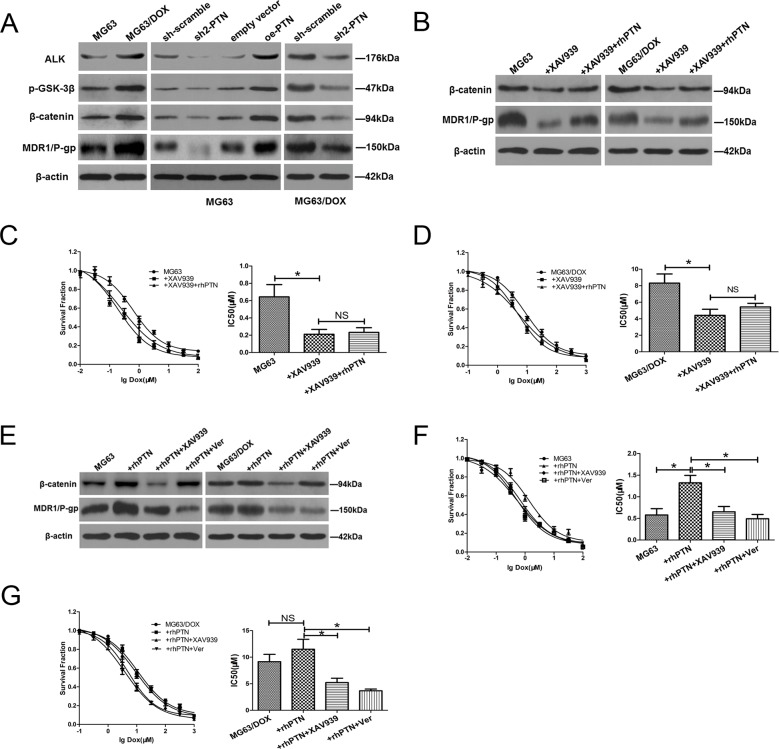
PTN promotes OS resistance to DOX by upregulating MDR1/P-gp through the ALK/GSK3β/β-catenin signaling pathway **(A)** The protein levels of ALK, β-catenin, MDR1/P-gp and p-GSK3β were higher in DOX-resistant MG63/DOX cells than in parental MG63 cells (left). PTN knockdown reduced the expression of these molecules in MG63 (middle) and MG63/DOX (right) cells, while PTN overexpression enhanced their expression in MG63 cells (middle) (β-actin was used as the reference protein). **(B)** β-catenin and MDR1/P-gp levels were reduced in MG63 (left panel) and MG63/DOX (right panel) cells treated with the β-catenin inhibitor XAV939, and supplementation with rhPTN could not reverse these effects (β-actin was used as the reference protein). **(C, D)** XAV939 impaired the chemoresistance to DOX in MG63 (C) and MG63/DOX (D) cells, and supplementation with rhPTN could not reverse this effect. **(E)** In rhPTN-treated MG63 (left) and MG63/DOX (right) cells, XAV939 reduced the expression of β-catenin and MDR1/P-gp, while the MDR1/P-gp inhibitor Verapamil only reduced the expression of MDR1/P-gp (β-actin was used as the reference protein). **(F, G)** rhPTN enhanced the resistance to DOX in MG63 (F) and MG63/DOX (G) cells, while XAV939 and Verapamil could equally reverse this effect. *, p<0.05. PTN: pleiotrophin; ALK: anaplastic lymphoma kinase; MDR1/P-gp: multidrug resistance protein 1/P-glycoprotein; OS: osteosarcoma; DOX: doxorubicin; rhPTN: recombinant human pleiotrophin; sh: short hairpin; oe: overexpression; NS: not significant.

Since aberrant activation of the β-catenin signaling pathway is a well-established molecular event driving the chemotherapy resistance of OS, we performed a series of mechanistic assays to determine whether this pathway is also responsible for PTN-induced resistance to DOX in OS cells [18, 19]. As shown in Figure 6A and Supplementary Figure 3A, Western blotting revealed that the expression of anaplastic lymphoma kinase (ALK), p-glycogen synthase kinase (GSK)3β, β-catenin and multidrug resistance protein 1/P-glycoprotein (MDR1/P-gp) was significantly higher in DOX-resistant MG63/DOX cells than in their parental MG63 cells. Moreover, PTN knockdown reduced the expression of ALK, β-catenin, MDR1/P-gp and p-GSK3β in MG63, MG63/DOX and U2OS cells, while PTN overexpression increased their expression in MG63 and U2OS cells. Therefore, we speculated that the ALK/GSK3β/β-catenin signaling pathway might be involved in PTN-induced chemotherapy resistance.

Then, we employed the β-catenin inhibitor XAV939 to suppress β-catenin activity in MG63 and MG63/DOX cells. As shown in Figure 6B and Supplementary Figure 3B, XAV939 effectively reduced the expression of β-catenin and MDR1/P-gp in OS cells. A chemosensitivity assay indicated that this inhibitor also dramatically reduced DOX resistance in MG63 (Figure 6C), MG63/DOX (Figure 6D) and U2OS cells (Supplementary Figure 3C). However, supplementation of XAV939-treated OS cells with rhPTN did not reverse the effects of XAV939 on β-catenin expression and chemotherapy resistance (also shown in Figures 6B-6D and Supplementary Figures 3B and 3C). Therefore, the ALK/GSK3β/β-catenin signaling pathway may be dominantly responsible for PTN-induced chemoresistance.

To explore the relationship between β-catenin and MDR1/P-gp further, we added the β-catenin inhibitor XAV939 or the MDR1/P-gp inhibitor Verapamil to rhPTN-treated OS cells. The addition of XAV939 reduced MDR1/P-gp expression, while Verapamil had no impact on β-catenin expression in rhPTN-treated MG63 and U2OS cells (Figure 6E and Supplementary Figure 3D). Functionally, XAV939 and Verapamil had similar neutralizing effects on PTN-induced chemotherapy resistance (Figures 6F and 6G and Supplementary Figure 3E). Together, these findings clearly demonstrated that PTN promotes OS chemoresistance to DOX by activating the ALK/GSK3β/β-catenin signaling pathway and upregulating MDR1/P-gp expression.

## DISCUSSION

Although DOX-based chemotherapy has dramat-ically improved the prognosis of OS patients during the past few decades, approximately 40-45% of patients have hardly benefited from it, largely due to their inherent chemotherapy resistance [20]. Researchers have made significant efforts to define the key molecular events causing chemotherapy resistance in OS, and microarray platforms have yielded numerous promising clinical biomarkers and targets [21]. In this study, based on the previous microarray studies of Mintz et al. and Walters et al. [16, 17], we not only identified PTN as a prognostic predictor for OS patients, but also demonstrated that PTN promotes OS resistance to DOX and determined the molecular mechanism.

As a heparin-binding growth factor, PTN was originally reported to be highly expressed in most embryonic organs, including the lung, gut and bone [6]. Recently, PTN overexpression has been detected in various tumor tissues, and may be clinically significant for cancer patients. For example, in gastric cancer, PTN overexpression was identified as an independent predictor of lower recurrence-free and overall survival [15]. Moreover, Jee et al. found that the PTN concentration was significantly increased in fine-needle aspiration samples from papillary thyroid cancer patients as compared with that in benign nodules, and might serve as a diagnostic/prognostic marker [22]. In this study, we found that PTN expression was associated with the local recurrence and necrosis rates of OS, although it was not significantly associated with other prognostic clinical features such as the TNM stage or lung metastasis. Since the local recurrence and necrosis rates are the main evaluation criteria for chemotherapeutic efficacy in OS, we speculated that PTN might be involved in the chemotherapy resistance of OS patients [23]. This speculation was confirmed by Kaplan-Meier survival analysis, in which overall survival and DFS were worse in OS patients with high PTN expression than in those with low PTN expression. In addition, univariate and multivariate analyses suggested that PTN expression is an independent adverse prognostic factor for DFS, strongly suggesting that detecting PTN expression may help to identify patients who are likely not to respond to standard chemotherapy and who will therefore need their therapeutic schedules altered.

Given that PTN expression clinically correlated with chemotherapeutic efficacy in OS patients, it was reasonable to hypothesize that PTN promotes the chemotherapy resistance of OS cells. To validate this hypothesis, we first employed shRNA or plasmid to down- or upregulate PTN expression in parental OS cells (MG63 and U2OS) and a DOX-resistant subline (MG63/DOX). PTN downregulation dramatically enhanced cellular susceptibility to DOX treatment *in vitro* and *in vivo*, while PTN upregulation had the opposite effect. Therefore, PTN promotes OS resistance to DOX treatment, a conclusion that accords somewhat with previous studies regarding the function of PTN in other cancer types. For example, in pancreatic cancer, PTN is a target of microRNA-137 and may promote cellular resistance to 5-fluorouracil treatment [24]. In breast cancer, there is also some evidence that PTN enhances resistance to anti-VEGF therapy by influencing immune cells [25]. Taken together, both our findings and those of previous studies confirm the clinical correlation of PTN with chemotherapy resistance, and also strongly suggest that PTN promotes OS chemoresistance to DOX treatment.

The ALK/GSK3β/β-catenin pathway is an impor-tant PTN-induced pathway that is involved in neural development and cancer progression [26–30]. PTN may also act as a ligand of ALK to maintain the cancer stem cell phenotype of glioblastoma [31]. Therefore, we foc-used on the ALK/GSK3β/β-catenin pathway in PTN-induced chemoresistance, and found that it was activated in DOX-resistant MG63/DOX cells. PTN downregulation restrained this pathway, while PTN upregulation enhanced it, suggesting that PTN positively regulates the ALK/GSK3β/β-catenin pathway. A subsequent functional assay with a β-catenin inhibitor (XAV939) further supported this conclusion; thus, we confirmed that PTN enhances OS resistance to DOX treatment by inducing the ALK/GSK3β/β-catenin pathway.

MDR1/P-gp, encoded by the *MDR1* gene, is a crucial promoter of chemoresistance, and its overexpression predicts poor clinical outcomes in OS patients [32]. Recent studies have indicated that MDR1/P-gp may be a downstream effector of β-catenin in the chemotherapy resistance of various cancer types, including colorectal, breast and ovarian cancer [33–35]. However, it has been unclear whether this protein is also a downstream effector of the ALK/GSK3β/β-catenin pathway in PTN-induced chemoresistance. To answer this question, we respectively added a β-catenin inhibitor (XAV939) and an MDR1/P-gp inhibitor (Verapamil) to rhPTN-treated OS cells. XAV939 and Verapamil both reversed the chemoresistance induced by rhPTN, implying that both β-catenin and MDR1/P-gp may be downstream effectors of PTN. Western blotting subsequently demonstrated that β-catenin inhibition reduced MDR1/P-gp expression, while MDR1/P-gp inhibition did not influence β-catenin expression, suggesting that MDR1/P-gp is positively regulated by β-catenin in PTN-induced chemoresistance. Based on these findings, we concluded that PTN promotes OS resistance to DOX by upregulating MDR1/P-gp through the ALK/GSK3β/β-catenin signaling pathway. This conclusion may also have some clinical significance for OS patients, because MDR1/P-gp overexpression is a well-known cause of OS chemoresistance, and there has been much effort to find effective approaches to reverse it [36, 37]. Our study has provided novel evidence that inhibitors of PTN could be developed as an alternative therapeutic strategy to inhibit MDR1/P-gp expression in OS patients who respond poorly to DOX-based chemotherapy regimens. We hope that this promising translation will be achieved and validated through future work integrating materialogy and pharmacology.

In conclusion, our study has demonstrated for the first time that PTN expression is an independent adverse prognostic factor for DFS in OS patients. *In vitro* and *in vivo* assays revealed that PTN enhances the chemoresistance of OS to DOX by activating the ALK/GSK3β/β-catenin signaling pathway and thus upregulating MDR1/P-gp expression. Therefore, PTN may be a reliable prognostic indicator for OS patients, and inhibitors of PTN could improve the chemotherapeutic efficacy in patients with unresponsive and recurrent OS.

## MATERIALS AND METHODS

### Patients and tissue samples

In total, 133 OS tissue samples were collected from patients who received radical surgery and standard preoperative chemotherapy (DOX, cisplatin, ifosfamide and high-dose methotrexate) at Shanghai Jiao Tong University Affiliated Sixth People's Hospital between November 2006 and November 2013. Based on their chemotherapeutic responses, patients were classified into the favorable group (≥90% tumor necrosis, Huvos III/IV grade) and the inferior group (<90% tumor necrosis, Huvos I/II grade) according to the criteria suggested by Huvos et al. [38]. All patients were pathologically diagnosed with OS by at least one experienced pathologist. Overall survival was calculated from the first neoadjuvant chemotherapy until death from any cause or the last observation. DFS was calculated as the time from the first neoadjuvant chemotherapy until tumor recurrence, metastasis or the last follow-up. This study was approved by the ethics committee of Shanghai Jiao Tong University Affiliated Sixth People's Hospital, and written informed consent was obtained from all patients to use their tissues for scientific research.

### Cell culture and reagents

MG63, SaoS2, U2OS, 143B and MNNG/HOS cells were obtained from the Chinese Academy of Sciences Cell Bank (Shanghai, China) and cultured in Dulbecco's modified Eagle medium (DMEM) (Invitrogen Life Technologies, Carlsbad, CA, USA) supplemented with 10% fetal bovine serum, penicillin (100 U/mL) and streptomycin (100 μg/mL) at 37°C in a 5% CO_2_ incubator. For the establishment of MG63/DOX, a DOX-resistant cell line, the parental DOX-sensitive MG63 cell line was cultured in media containing increasing concentrations of DOX (from 5 nM to 100 nM) over six months. The resistant cells were incubated in DOX-free medium for at least seven days prior to the assays.

DOX, XAV939 and Verapamil were purchased from Selleck Chemicals (Houston, TX, USA) and dissolved in dimethyl sulfoxide (DMSO) (maximum concentration: 0.2%, Sigma-Aldrich, St. Louis, MO, USA) as a stock solution. rhPTN was obtained from PeproTech (Rocky Hill, NJ, USA).

### Quantitative RT-PCR analysis

Total RNA was extracted with TRIzol reagent (Invitrogen Life Technologies) and reverse-transcribed to cDNA with a Prime-Script RT-PCR Kit (Invitrogen Life Technologies). The forward primer 5′-GGGGAGAATGTGACCTGAAC-3′ and reverse primer 5′-AGGGCTTGGA GATGGTGA-3′ were used for *PTN*. The results were normalized to *β-actin* levels, which were determined with the following primers: forward 5′-GTGGACATCCGCAAAGAC-3′ and reverse 5′-AAAGGGTGTAACGCAACTA-3′. The reactions were performed with a SYBR Green Kit (Takara, Japan) and the relative mRNA levels were calculated by the 2^−ΔΔCt^ method.

### Lentiviral construction and infection

Three small shRNAs (sh1: 5′-AGGAGAAG ATGCTGGATTA-3′, sh2: 5′-TCAAGCAGAAT CTA AGAAG-3′; sh3: 5′-TGGAGCTGAGTGCAAGCAA-3′) targeting *PTN* were inserted into a plasmid, and the knockdown efficiency in OS cells was verified by Western blotting. The shRNA exhibiting the best knockdown efficiency was chosen for lentiviral development, and a non-silencing scrambled shRNA was used as a negative control (5′-UUCUUCGAACGUGUCACGUTT-3′). The lentiviruses were constructed routinely, as described previously [39]. In brief, Lipofectamine 2000 (Invitrogen Life Technologies) was used to transfect 293T cells with shRNA-expressing vectors (or *PTN*-expressing cDNAs) and virion-packaging elements (pCMV 4R8.92 and pVSVG-I). The lentiviral shRNAs and cDNAs were transfected into OS cells. Pools of stable transductants were generated by selection with 3 μg/mL puromycin hydrochloride (Sigma-Aldrich) for four weeks and expanded in 1 μg/mL puromycin.

### Chemosensitivity assay

Chemosensitivity assays were performed with Cell Counting Kit-8 (CCK-8, Dojindo Laboratories, Shanghai, China) according to the manufacturer's instructions. Cells were seeded into 96-well plates at 4×10^3^ cells/well and incubated with complete medium at 37°C. After cellular adherence, graded concentrations of DOX were added to the medium for 48 h, and then the cells were incubated with CCK-8 solution for 90 min. Finally, the optical density of the cells was measured at 450 nm on a microplate reader (Bio-Rad, Hercules, CA, USA). The cellular sensitivity to DOX was evaluated by the IC50, the concentration that inhibited cell growth by 50%.

### Cellular apoptosis and colony formation assay

For apoptotic analysis, transfected OS cells were seeded into six-well plates at a density of 2×10^5^ cells/well and harvested after incubation with or without 0.1 μM DOX for 48 h. Then, cells were double-stained with Annexin V-allophycocyanin (APC) and 7-aminoactinomycin D (KeyGen Biotech, Nanjing, China), and analyzed on a flow cytometer (BD Biosciences, San Jose, CA, USA).

For the colony formation assay, 400 transfected OS cells/well were seeded into six-well plates in medium with or without 0.1 μM DOX. After incubation for 12 days, cells were fixed with paraformaldehyde and stained with 0.1% crystal violet dye (Sigma-Aldrich). Finally, the number of colonies (consisting of at least 50 cells) was counted under an optical microscope.

### Western blot analysis

Cells were lysed in radioimmunoprecipitation assay (RIPA) buffer in the presence of protease inhibitors (Complete mini, Roche, Indianapolis, IN, USA). The protein concentration was determined with the BCA protein assay kit (Beyotime Biotechnology, Beijing, China), and equal amounts of protein were separated by SDS-PAGE. Then, the proteins in the gels were transferred to polyvinylidene difluoride membranes and blocked with TRIS-buffered saline containing 5% bovine serum albumin. The blots were probed with primary antibodies to PTN (1:1000, Abcam, Cambridge, MA, USA), ALK (1:2000, Abcam), phospho-GSK3β at serine 9 (1:500, Abcam), β-catenin (1:4000, Abcam), MDR1/P-gp (1:500, Proteintech, Chicago, IL, USA) and β-actin (1:2000, Abcam) overnight at 4°C. Blots were then probed with a horseradish peroxidase-conjugated secondary antibody (1:5000, Santa Cruz Biotechnology, Dallas, TX, USA) for 90 min at room temperature before visualization with an enhanced chemiluminescence detection system.

### Xenograft models

Twenty-four athymic male BALB/c nude mice (four to five weeks old) were housed under specific-pathogen-free conditions and randomly divided into four groups (n=6). Transfected OS cells in the logarithmic phase were harvested and suspended in 200 μL serum-free medium at a concentration of 2.5×10^6^ cells/mL. The suspension was then subcutaneously injected into both groins of each nude mouse. Nine days after the injection, normal saline or DOX (5 mg/kg) was injected subcutaneously into each mouse twice per week. Tumor growth was monitored every three days with a caliper and was calculated with the following formula: volume= 0.5×length×width^2^. Twenty-seven days after the injection, the mice were sacrificed and the tumors were harvested. All procedures were performed according to protocols approved by the Animal Care and Use Committee of Shanghai Jiao Tong University Affiliated Sixth People's Hospital.

### Immunohistochemistry

Sections 4-μm thick were cut from paraffin-embedded specimens, deparaffinized and boiled in 0.01 mol/L citrate buffer (pH 6.0) in a microwave for 20 min. The sections were then immersed in 0.3% hydrogen peroxide following washes with phosphate-buffered saline (PBS). The sections were incubated with a primary antibody against PTN (1:50, Abcam) at 4°C overnight. Then, the sections were incubated with polyclonal goat anti-rabbit IgG (1:250, Abcam) at 37°C for 20 min. After being washed with PBS, sections were incubated with diaminobenzidine and counterstained with hematoxylin. Sections incubated with PBS instead of the primary PTN antibody were used as negative controls.

The staining evaluation was conducted by two researchers who were blinded to the patients’ clinicopathological information. The evaluation principle was based on the IRS, defined as the percentage of positive cells score multiplied by the staining intensity score. The percentage of positive cells was scored as follows: 0 (<5%), 1 (5–25%), 2 (25–50%), 3 (50–75%), and 4 (>75%). The staining intensity was scored as follows: 0 (negative), 1 (weak), 2 (moderate), and 3 (strong). An ROC curve was employed to determine the cutoff value of the IRS, as described previously [40]. Sections with scores greater or less than the cutoff value were regarded as having high or low expression, respectively.

### Statistical analysis

SPSS 21.0 statistical software (SPSS, Chicago, IL, USA) was used for statistical analyses, and data are presented as the mean±standard deviation. Correlations between PTN expression and clinicopathological parameters were evaluated with Pearson's chi-squared test. A Kaplan-Meier survival model was used to depict the survival curves, and a log-rank test was used to determine intergroup differences. Univariate and multivariate analyses based on the Cox proportional hazards regression model were used to identify independent prognostic factors affecting patients’ overall survival/DFS. For *in vitro* and *in vivo* experiments, two-tailed Student's t tests were used to evaluate the statistical significance of differences between two groups. A P value < 0.05 was considered to be statistically significant.

## SUPPLEMENTARY MATERIALS FIGURES


