# Supplementary material for: Pleiotrophin promotes chemoresistance to doxorubicin in osteosarcoma by upregulating P-glycoprotein
Source: Oncotarget. 2017 Jul 10;8(38):63857–70. doi: 10.18632/oncotarget.19148 (PMC5609967; doi:10.18632/oncotarget.19148)
Supplement: Supplementary file 1 [file oncotarget-08-63857-s001.pdf]

Pleiotrophin promotes chemoresistance to doxorubicin in osteosarcoma by upregulating P-glycoprotein

SUPPLEMENTARY MATERIALS

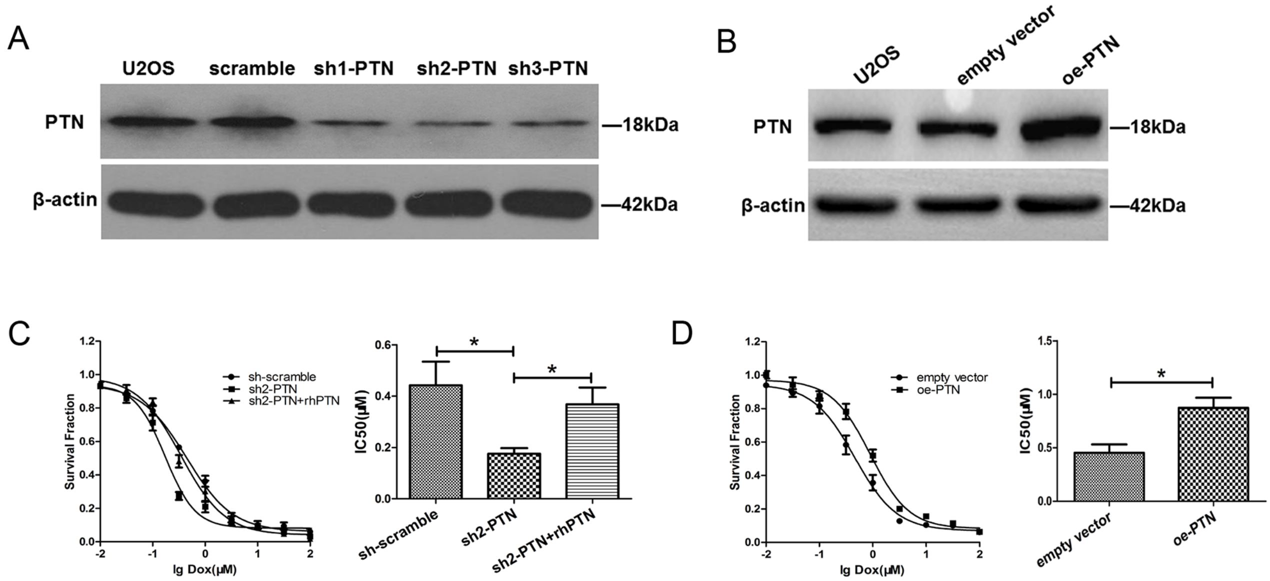

Supplementary Figure 1: PTN promotes DOX resistance in U2OS cells. (A) The knockdown efficiency of three shRNAs targeting PTN was examined in U2OS cells by Western blotting. **(B)** The efficiency of the PTN overexpression plasmid was examined in U2OS cells by Western blotting. **(C)** PTN knockdown inhibited DOX resistance in U2OS cells, while supplementation with rhPTN for 24 h (100 ng/mL) reversed this effect. **(D)** PTN overexpression promoted DOX resistance in U2OS cells. *$p < 0.05$. PTN: pleiotrophin; DOX: doxorubicin; rhPTN: recombinant human PTN; sh: short hairpin; oe: overexpression.

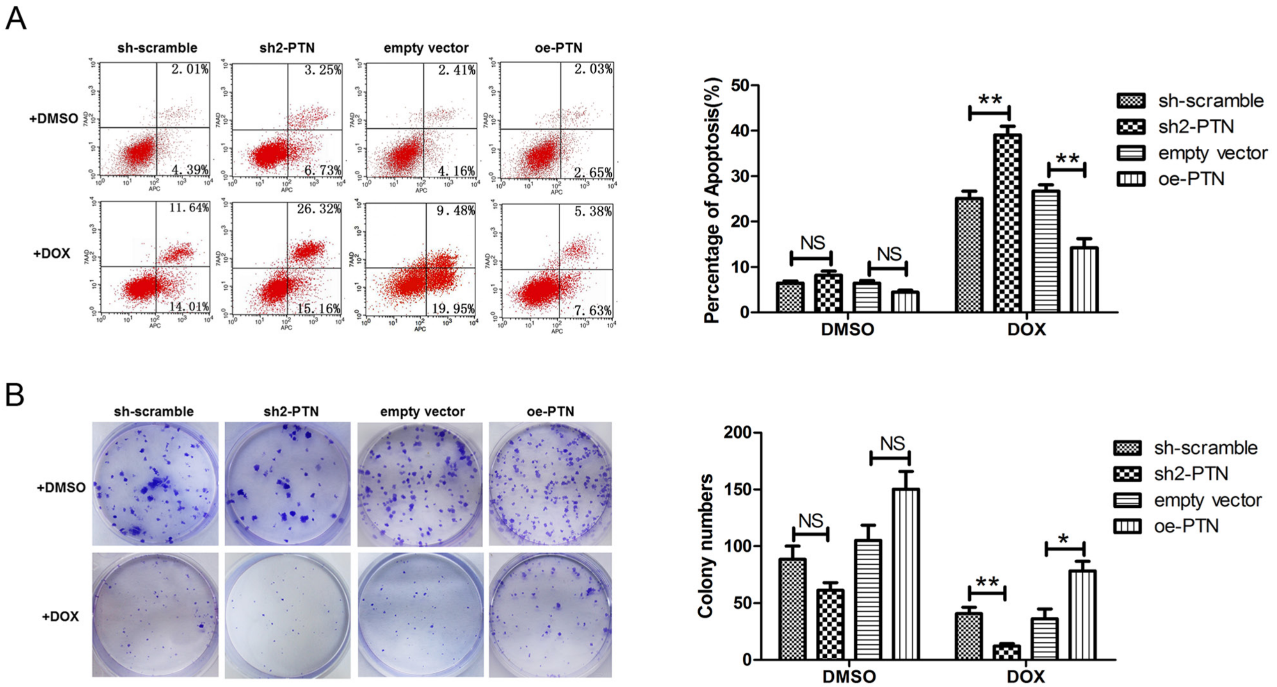

Supplementary Figure 2: PTN promotes apoptosis resistance and colony formation in U2OS cells treated with DOX. **(A)** PTN expression had no effect on the apoptosis of U2OS cells treated with DMSO. PTN knockdown promoted apoptosis in U2OS cells treated with DOX, while PTN overexpression inhibited their apoptosis. **(B)** PTN expression had no effect on the clone formation of U2OS cells treated with DMSO. PTN knockdown inhibited the clone formation of U2OS cells treated with DOX, while PTN overexpression enhanced it. *, p<0.05; **, p<0.01. PTN: pleiotrophin; DOX: doxorubicin; sh: short hairpin; oe: overexpression. NS, not significant.

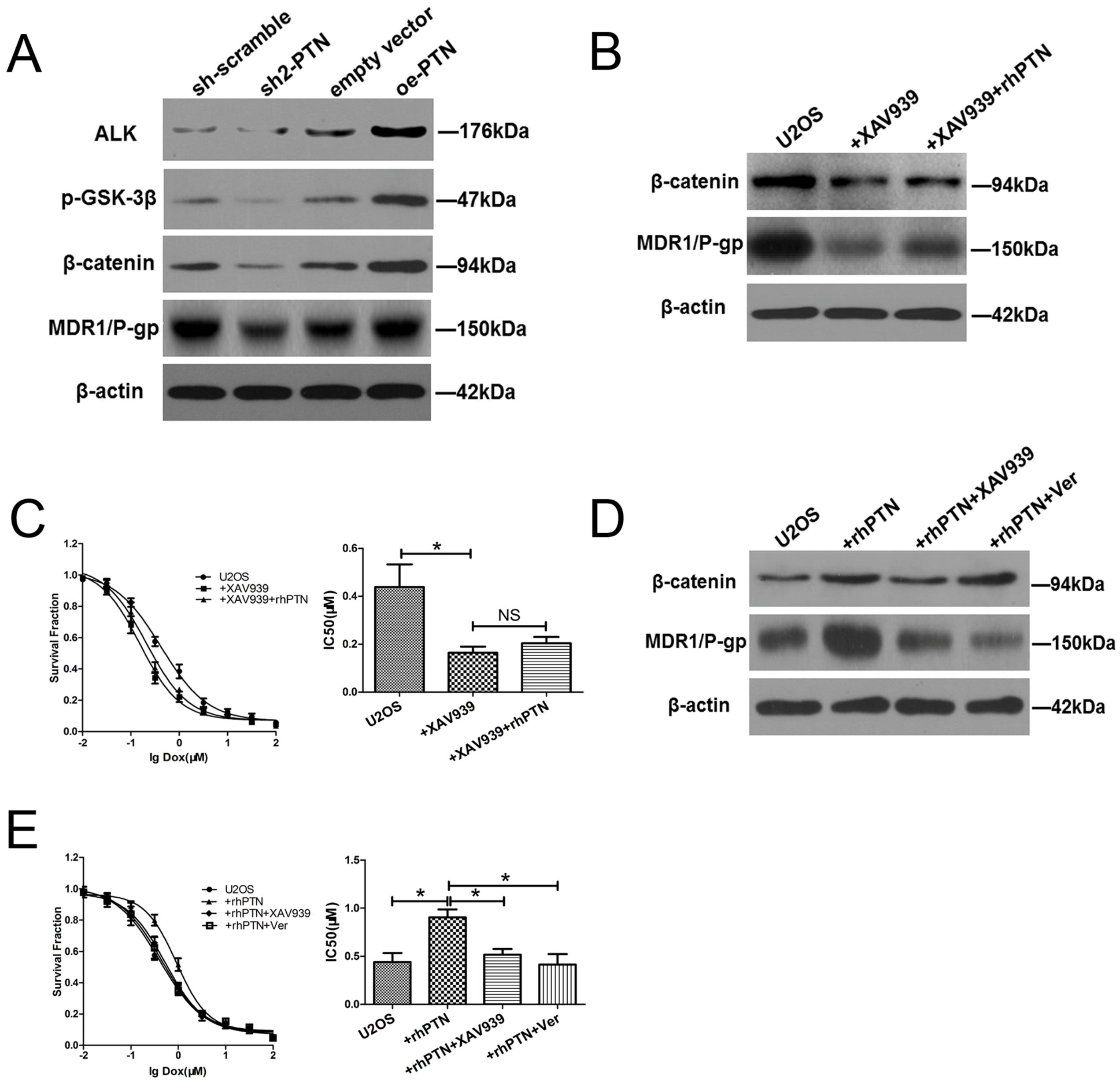

Supplementary Figure 3: PTN promotes DOX resistance by upregulating MDR1/P-gp through the ALK/GSK3β/β-catenin signaling pathway in U2OS cells. (A) PTN knockdown reduced the expression of ALK, β-catenin, MDR1/P-gp and p-GSK3β in U2OS cells, while PTN overexpression enhanced their expression (β-actin was used as the reference protein). **(B)** β-catenin and MDR1/P-gp levels were reduced in U2OS cells treated with the β-catenin inhibitor XAV939, and these effects could not be reversed by supplementation with rhPTN (β-actin was used as the reference protein). **(C)** XAV939 impaired the chemoresistance to DOX in U2OS cells, and this effect could not be reversed by supplementation with rhPTN. **(D)** In rhPTN-treated U2OS cells, XAV939 reduced the expression of β-catenin and MDR1/P-gp, while the MDR1/P-gp inhibitor Verapamil only reduced the expression of MDR1/P-gp (β-actin was used as the reference protein). **(E)** rhPTN enhanced DOX resistance in U2OS cells, while XAV939 and Verapamil equally reversed this effect. *, $p<0.05$. PTN: pleiotrophin; ALK: anaplastic lymphoma kinase; MDR1/P-gp: multidrug resistance protein 1/P-glycoprotein; DOX: doxorubicin; rhPTN: recombinant human PTN; sh: short hairpin; oe: overexpression; NS, not significant.
